# Systematic review of phenotypes in McLeod syndrome and case report of a progressive supranuclear palsy in a female carrier

**DOI:** 10.1186/s13023-024-03309-4

**Published:** 2024-08-25

**Authors:** Andreas Albert Braun, Hans Heinrich Jung

**Affiliations:** Department of Neurology, University Hospital Zurich, Frauenklinikstrasse 26, 8091 Zurich, Switzerland

**Keywords:** Progressive supranuclear palsy, McLeod syndrome, Neuroacanthocytosis, Systematic review, Phenotype

## Abstract

**Introduction:**

We present a systematic review of phenotypes of McLeod syndrome (MLS) and a case of a 73-year-old female carrier of the genetic alteration leading to MLS with the typical progressive supranuclear palsy (PSP) phenotype.

**Methods:**

To facilitate clinical reasoning and enable targeted diagnosis, we conducted a systematic review of the papers describing symptomatic cases of confirmed McLeod syndrome. This review follows the PRISMA 2020 statement: an updated guideline for reporting systematic reviews (Page et al in Syst Rev 10(1):89, 2021).

**Results:**

The average onset of MLS was at 40.2 years of age with chorea (46%), seizures and psychiatric changes (each 13%). Very common are weakened Kell antigen (100%), changes in muscle biopsy (100%), genetic alterations in XK (100%), elevated creatine kinase (97%), acanthocytes (96%), MRI changes (95%), chorea (84%) and hyporeflexia (82%).

**Conclusion:**

This review of 65 males and 11 females gives a concise overview of clinical phenotypes in MLS, reinforcing our view that this female patient had PSP independent of MLS carrier status. This report highlights the pitfalls of anchoring in medical decision-making, particularly the possible diagnostic bias of a known genetic carrier status of a very rare disease.

## Background

Neuroacanthocytosis is a group of rare genetically determined diseases characterized by movement disorders and red blood cell acanthocytosis. This group encompasses autosomal recessive chorea-neuroacanthocytosis (ChAc) and X-linked McLeod syndrome (MLS) with mutations in the *VPS13A* gene and the *XK* gene, respectively. Since VPS13A and XK proteins interact, the two disorders are called “VPS13A-opathies” [2].

MLS and ChAc resemble the chorea of Huntington’s disease and are characterized by psychiatric symptoms, cognitive impairment and cardiopathy. Distinguishing features are head dropping, rubber man-like gait, tongue protrusion dystonia, and tongue and lip biting, the latter particularly in ChAc. Neuromuscular involvement with areflexia and neurogenic and myopathic muscle alterations are common in these two diseases, and patients with MLS may develop cardiac disease, which constitutes the cause of death in approximately 50% of cases [3].

Whereas autosomal recessive ChAc usually manifests between 20 and 30 years of age, men with X-linked MLS usually develop neurological symptoms between 25 and 60 years of age, displaying the typical phenotype mentioned above [4].

While ChAc can lead to vertical gaze impairment, there is no such case described in MLS [5]. The only mention of PSP in MLS is: “He developed marked progressive parkinsonian features with a masked facies and reduced blinking, frontalis contraction similar to that observed in progressive supranuclear palsy, hypophonic and monotonous speech, rigidity, and generalized bradykinesia.” [6].

## Case report

A neurologist of a private practice referred a 73-year-old woman to our department because of a “myopathic syndrome with myopathic facial expression, proximal pareses and slight dysarthria in the context of McLeod syndrome” for a second opinion.

In June 2023, the patient complained about progressive symptoms for five years with deterioration in the last two years. She was depressed, easily fatigued, dropped objects, suffered from recurrent falls backwards, had problems to swallow solid food and often stopped in the process of getting clothed. Her memory function deteriorated, she spoke monotonously, was sensitive to light and developed a slight hand tremor. The depression had improved with Bupropion. She took gingko medication to support cognition, perindopril/indapamid for hypertensive cardiopathy, as well as trazodone and quetiapine for sleep.

In 2005, the patient had already come for genetic counseling to our department. Kell positive and negative erythrocytes had been found in the flow cytometry, so that her carrier status for McLeod-Syndrome (MLS) had been confirmed without genetic testing.

**Fig. 1:**
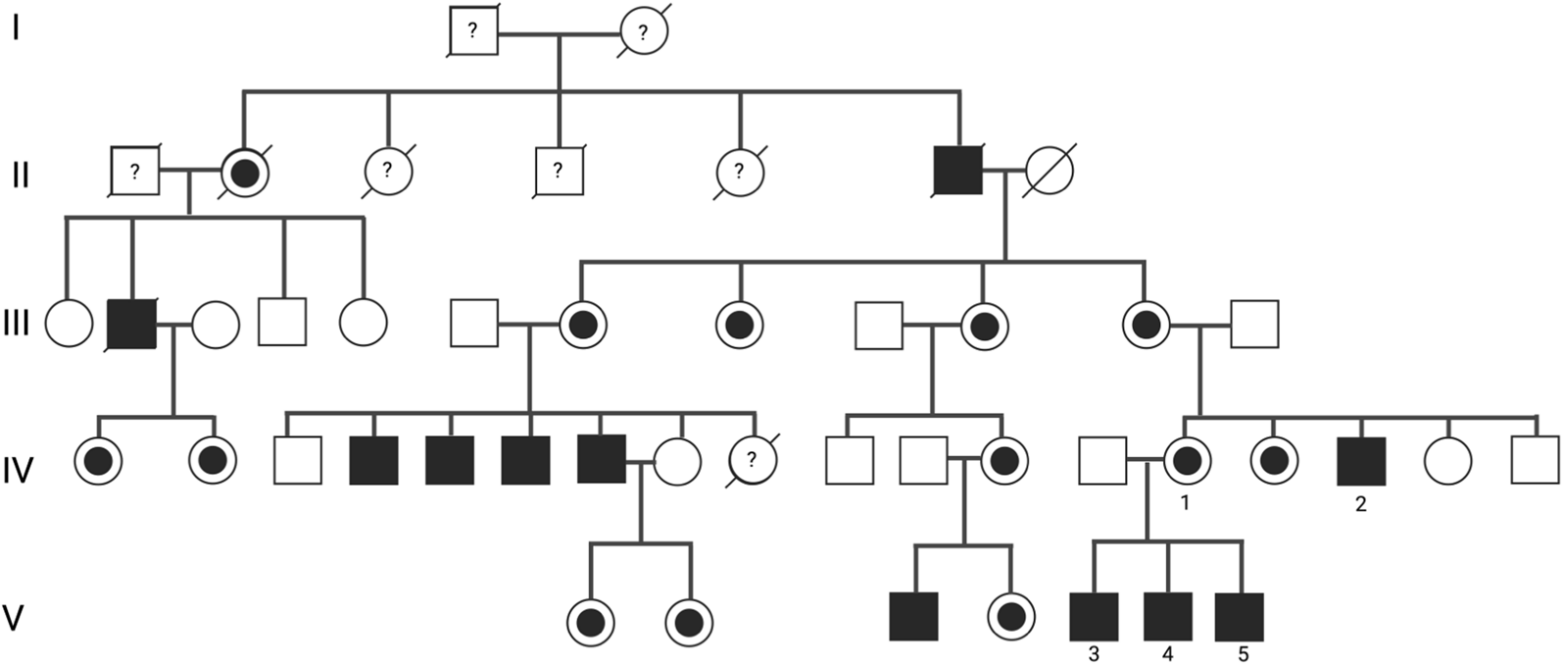
Pedigree of the largest McLeod family described in literature. IV-1 is the patient of the case report, IV-2 the brother, V-3, V-4, V-5 the three children. [7] Modified from Jung et al. with permission of the author

**Fig. 2:**
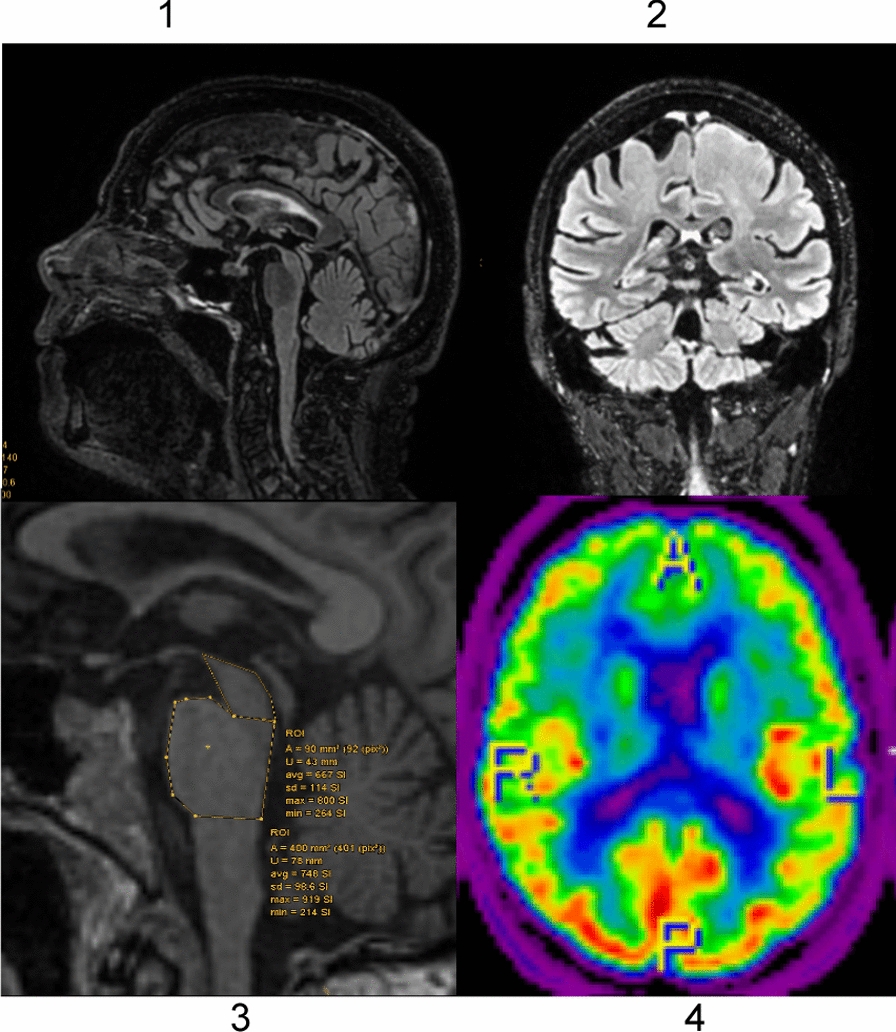
1–4. 1–3: FLAIR MRI, 4: FDG-PET. 1: sagittal, hummingbird sign. 2: coronar, atrophy of superior cerebellar peduncle 3: sagittal, pons-midbrain ratio of 0.225.4: axial, high metabolism occipital, low metabolism frontal and in caudate nucleus

**Fig. 3:**
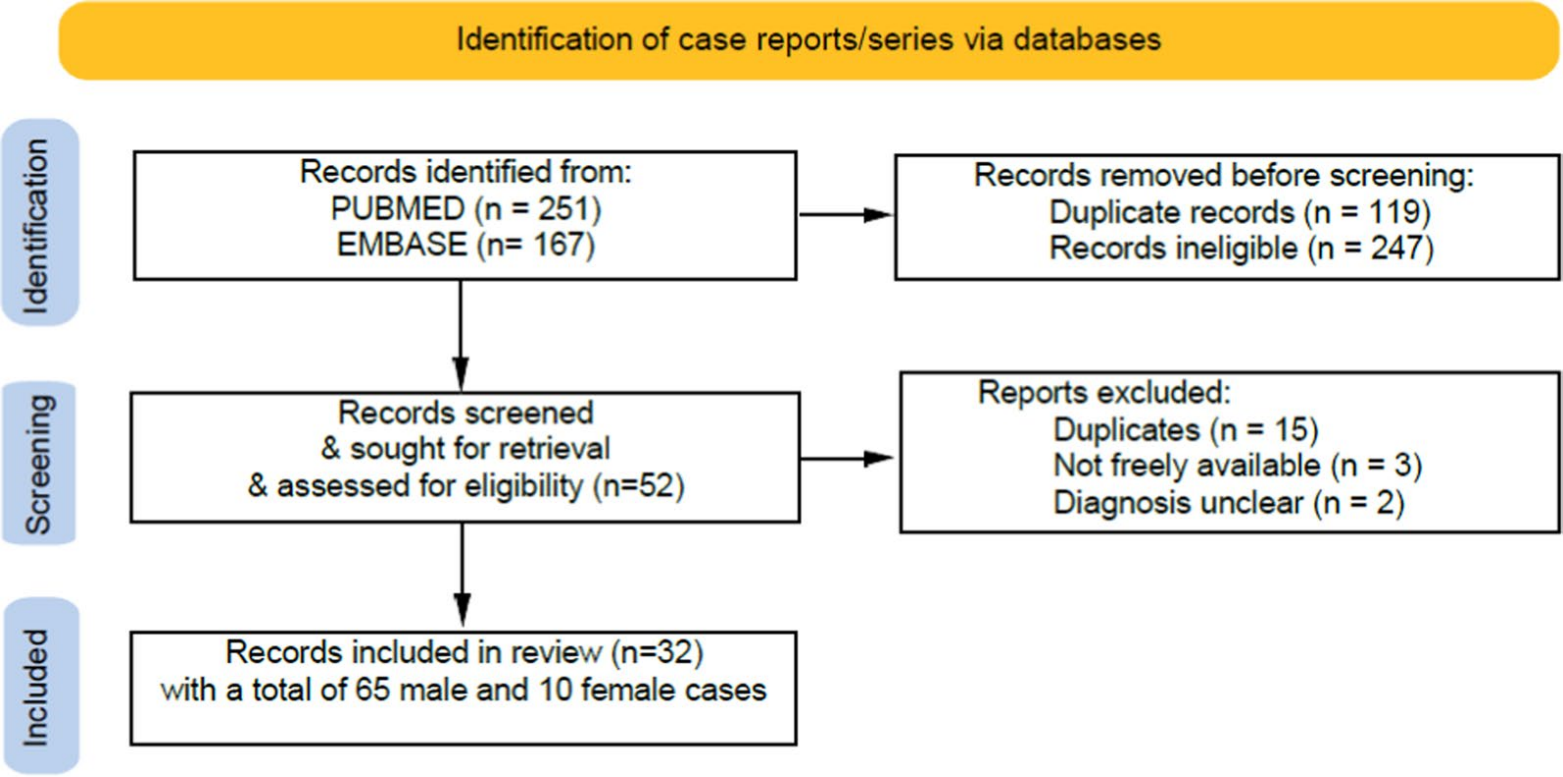
PRISMA flowchart of the process for the identification of records [9]

**Fig. 4:**
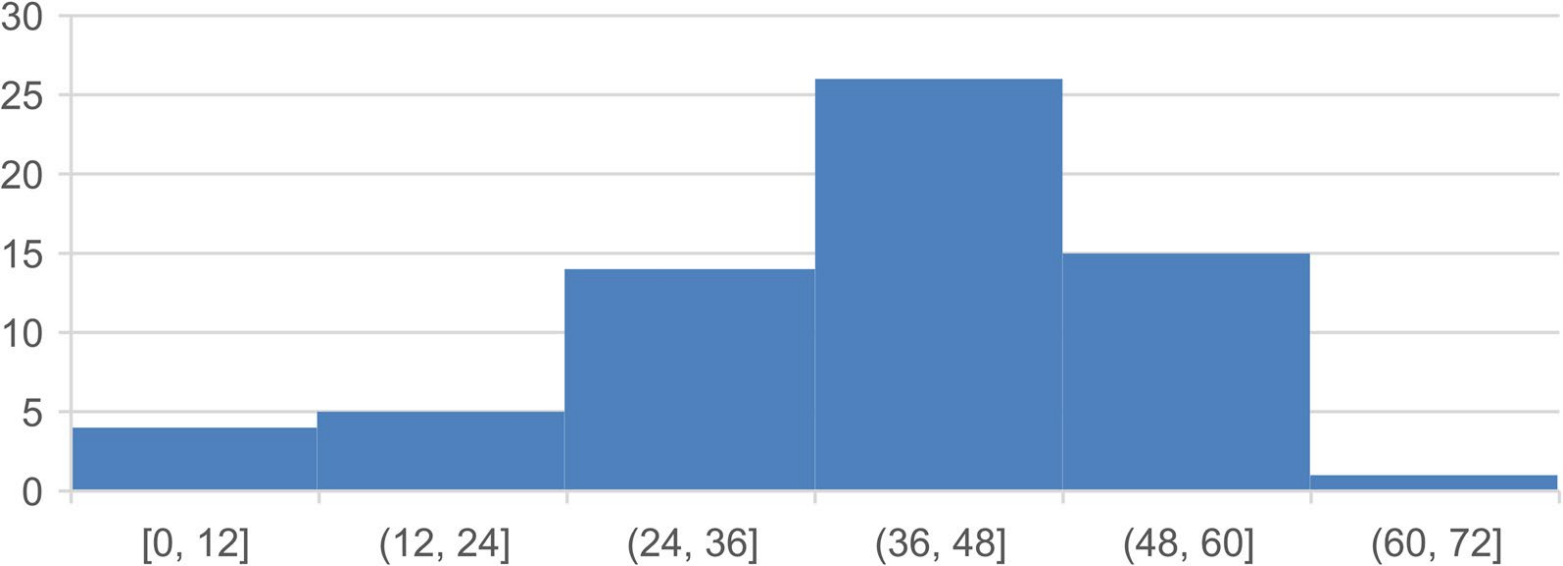
Age of onset in male patients with MLS. X-axis age in years in blocks of 12 years, y-axis percent of total, number = 63

**Fig. 5:**
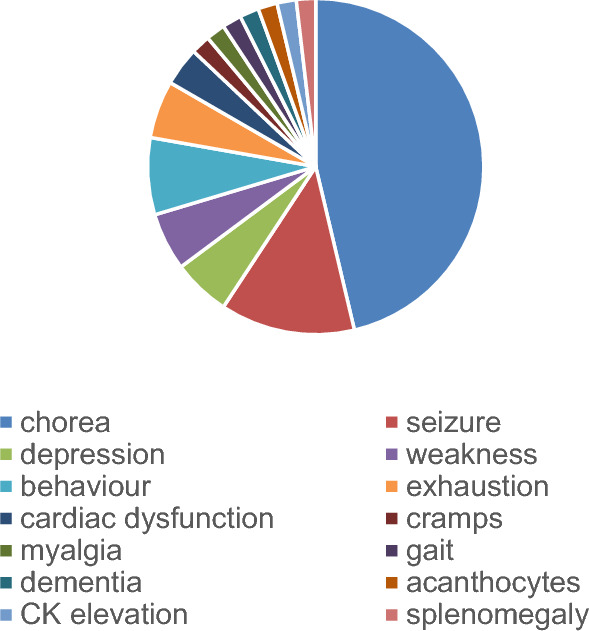
First symptom reported in male patients with MLS as a pie chart

Family history (Fig. 1) was remarkable for a brother (Fig. 1, IV-2) with MLS having a mutation in Q299X of the XK gene and an asymptomatic mother carrying the same mutation. Part of the family had been previously described [7]. The patient had three sons born in the 70 s. The oldest son (Fig. 1, V-3) had already developed compulsive-obsessive symptoms as a child about hiding his gynecomastia, compulsive hand washing, showering and floor cleaning. He urinated in public and stole vegetables in stores. He took several sedatives in an appellative intention. The Q299X mutation was confirmed. The second son (Fig. 1, V-4) had some choreatic intrusions and a “choppy” gait. The youngest son (Fig. 1, V-5) seemed always nervous and his legs had choreatic movements.

Neurological examination revealed psychomotor slowing, hypomimia, dysarthria, reduced postural stability, vertical gaze palsy, slowed horizontal saccades, bradydysdiadochokinesia, slight rigidity on both sides and slight proximal and distal arm weakness. The ankle jerk reflex was reduced compared to the brisk reflexes of the arms.

Neuropsychological testing demonstrated frontosubcortical deficits. Creatine kinase (132 U/L) and cerebrospinal fluid (including Tau and beta-amyloid) were normal. EMG showed signs of mild myopathy but also chronic neurogenic alterations.

MRI revealed slight global atrophy and leukoencephalopathy and a pathological score of the magnetic resonance parkinsonism index. FDG-PET showed reduced metabolism in the basal ganglia and frontal ganglia.

Conclusively, we diagnosed PSP because of the typical findings. At least her tremor improved with levodopa, while she otherwise progressed (Fig. 2).

## Systematic review of phenotypes in McLeod neuroacanthocytosis syndrome

### Methods

We started with medical subject headings (MeSH) about McLeod Syndrome and neuroacanthocytosis and refined them with the Yale MeSH Analyzer [9]. We soon abandoned this approach because MLS is so rare and its name unique that a narrowing of search results was not necessary. We identified 251 PubMed and 167 Embase listed papers containing “McLeod syndrome”. We considered all articles containing symptoms and signs in McLeod neuroacanthocytosis syndrome on an individual level (case reports, case series or stating exact numbers of cases with references to check for double entries). Confirmation of the diagnosis is defined as typical abnormalities in the Kell system, positive genetic testing or typical and specific symptoms (e.g. chorea) and a diagnosed family member. Only 32 records fulfilled these criteria (s. flowchart) because most articles did not state information about individuals or appeared in either databank. Eighteen PUBMED articles were excluded because of already described patients in other papers, no open access, incidental finding without typical symptoms (such as a febrile seizure during infancy) or unclear diagnosis (MLS versus autosomal-recessive chorea-acanthocytosis). The search on Embase only identified 4 records from 2023 that were not on PubMed, of which two had to be excluded (MLS already known in 1 patient, no neurological information given and no genetic or Kell testing done in the other).

One reviewer assessed the title and abstract of potentially eligible articles, accessed the relevant articles and created a table with Microsoft Excel 2016 containing the prominent features of each case. Another reviewer double-checked the entries.

In summary, we identified 75 patients, 65 males and 10 females (plus our case). Only 6 females had specific symptoms of MLS (chorea), and only two were genetically proven to be susceptible to the disease by skewed X inactivation.

We extracted the information that can be seen in Table 1. We calculated the prevalence of these findings. The most important decision was how to treat missing entries. We judged that the absence of reported parameters such as liver enzymes might be either an omission to mention or a negative value, so a range was calculated based on the denominator of all 65 male patients or 65 minus the missing values. Other parameters, e.g. a brain MRI or a genetic testing would have probably been stated even in the context of negative findings so that a missing report decreases the denominator.

The data collection process took place in July 2023 and analysis until August 2023. This research did not receive any specific grant from funding agencies in the public, commercial, or not-for-profit sectors.

## Results

Figure 3 shows the flowchart of identification, screening and inclusion of cases. The results of female patients are shown for each patient in Table 1, and the results for male patients are shown cumulatively in Table 2.

**Table 1:** Phenotypes of female heterozygous patients with McLeod syndrome (MLS)

Patients	Source
II.1: Onset at age 51, with chorea, hyporeflexia, CK 840 U/L, acanthocytes 25%, skewed X-inactivation, neuronal loss of basal ganglia, a 1-bp deletion in exon 2 at codon 90 of the XK gene II.2: Chorea of lower limbs and areflexia, X-inactivation skewing at the upper limit of normal	[10]
1 sister of index patient with involuntary movements (“Tic-like”)	[11]
3 females in MLS family with cognitive deficits, 2 below 50 years of age. None of them had other signs such as areflexia, acanthocytes or elevated CK. Three other of 15 females had acanthocytosis (8–12%), one had elevated CK (360 U/L), two female mutation carriers also had a significant reduction of striatal FDG uptake	[7]
The mother of case 4 has impaired balance, generalized chorea, memory loss, polyneuropathy 1 aunt of case 4 with cognitive impairment	[12]
2 female family members of a patient with mild involuntary movements	[13]
Our case summarized: Slight pareses of arms, weak Kell expression, myopathic and neurogenic changes on EMG probably due to MLS. The changes would probably have gone unnoticed without thorough examination in the context of the PSP phenotype as described above

**Table 2:** Cumulative findings in male patients with McLeod neuroacanthocytosis syndrome. Sources: [3, 6, 7, 12–40]

Finding	n	%	Comments
Remarkable family history, n = 42	25	38–60%	Often not mentioned at all. Therefore, calculated with denominator of 42 (explicitly mentioned) and 65 (all)
Chorea, n = 61	51	84%	48 generalized, 2 legs, 1 only facial
Weakness, n = 56	30	54%	Exact location rarely mentioned. The few imaging findings and clinical descriptions showed rather proximal atrophies and fatty degeneration. There is probably a weakness in most patients in the course of the disease. Otherwise, described as leg and distally dominant [40]
Parkinsonism, n = 65	8	12%	Bradykinesia, dysdiadochokinesia
Dystonia, n = 65	4	6%	Except feeding dystonia (see below). Most likely, underreported
Gait abnormalities, n = 65	12	18%	Mostly through hyperkinesia and possibly neuropathy
Seizures, n = 56	17	30%	Only generalized seizures described
Dysarthria, n = 48	21	44%	Rather underestimated, some patients with strong facial hyperkinesia or feeding dystonia were characterized as not having dysarthria
Tongue/lip biting, n = 65	9	14%	Often mentioned in the context of Chorea-acanthocytosis. However, with 14% not uncommon in MLS
Vocalization, n = 65	11	17%	Grunting, belching, Tourette-like
Feeding dystonia, n = 65	3	5%	i.e., sticking out the tongue during feeding
Psychiatric disorder, n = 65	31	48–60%	Depression, restlessness, anxious, compulsive-obsessive, disinhibition. Range depending on denominator (52 vs. 65)
Cognitive, n = 65	32	49–62%	Mainly frontosubcortical or mnestic deficits. Range depending on denominator (52 vs. 65)
Hyporeflexia, n = 55	45	82%	In 7 patients explicitly stated more reduced in the legs
MRI changes, n = 40	38	95%	72.5% had atrophy of basal ganglia, 20% general atrophy, 12.5% white matter lesions
Neuropathy, n = 38	35	53–92%	4 only clinically diagnosed, 1 demyelinating, 17 axonal, 3 times myopathy on EMG. Often not mentioned at all. Therefore, calculated with denominator of 38 (explicitly mentioned) and 65 (all)
Cardiopathy, n = 52	28	55%	Mostly not specified, 5 times dilated, 1 hypertrophied, 1 arrhythmic
CK elevated, n = 61	59	97%	average 1473 U/L, range from normal to 4238 U/L
Liver enzymes elevated (AST, ALT, GGT), n = 65	31	37–77%	ALT (hepatospecific) equally or more elevated than AST (which is also in muscle cells) as an indication that the enzymes do derive from hepatocytes rather than muscle cells. Depending on denominator 31 vs. 65 (all). Maximum was 6 times upper limit of normal
LDH elevated, n = 65	8	12%	Often not mentioned at all. Therefore, this value reflects the minimum. Maximum was 527 U/L
Acanthocytes, n = 55	53	96%	On average 17% acanthocytes, range 3–85%
Kell anomaly, n = 61	61	100%	Reduced or absent Kell antigen or Kell positive and negative populations
Anemia, n = 65	6	9%	Often compensated hemolysis. In other study 80% reduced haptoglobin [3]
Genetics XK, n = 52	52	100%	Heterogeneous. Frameshift, missense and nonsense mutation and deletions described
Hepatomegaly, n = 65	7	11%	Often not mentioned. This value rather reflects the minimum
Splenomegaly, n = 65	5	7.6%	Often not mentioned. This value rather reflects the minimum
Muscle biopsy, n = 12	12	100%	Mostly biopsy of quadriceps, 2 myopathic, 4 mixed, 6 neuropathic

Only 6 females are likely to have had MLS because of more specific findings, such as chorea. A skewed X-inactivation was proven in 2 patients. Our patient had the typical but not very frequent and not specific combination of neurogenic and myopathic changes on EMG and may have a mild muscle manifestation of MLS that would have gone unnoticed without the PSP (Table 1).

In addition to the results in Table 2, the following symptoms and signs were found in male patients: dysphagia (2), tremor (2), insomnia (2), abnormal saccades (2), bruxism (1), rubber-man-like appearance (1), ataxia (1), apraxia (1), rhabdomyolysis (1) and respiratory failure (1).

The age of onset (n = 62) had a mean of 40.2 years of age (y) (median 41.5 y), with an SD of 11.4 y and an interquartile range of 34–49 y. The onset ranges from childhood to 61 y in this slightly to the left skewed graph (Fig. 4).

As seen in Fig. 5, the first symptoms in male patients with MLS were chorea (46%), followed by seizures (13%), psychiatric changes (behavior alterations, depression) in 13% and weakness, exhaustion (each 5.6%) and cardiac dysfunction (3.7%). Other symptoms appeared once each (1.9%).

## Discussion

The first clinical manifestations of McLeod syndrome (MLS) in men start on average at 40.2 years of age. Almost half of the patients’ first symptom was chorea (46%), followed by seizures and psychiatric changes (each 13%). The most common findings in MLS are weakened Kell antigen (100%), genetic alterations in XK (100%), changes in muscle biopsy (100%, 4 myopathic, 6 neurogenic alterations, 4 mixed), elevated creatine kinase (97%), acanthocytes (96%), MRI changes (95%, mainly atrophy of basal ganglia), chorea (84%) and hyporeflexia (82%). A total of 52–93% of patients had signs of neuropathy. Up to two-thirds of the patients had psychiatric (depression, compulsion, disinhibition) or cognitive changes (fronto-subcortical, memory). Almost half of the patients had dysarthria, and more than half of the patients had a diagnosis of cardiopathy. One-third had tonic‒clonic seizures. Other findings are elevated liver enzymes, LDH, anemia, hepatosplenomegaly, involuntary vocalization or feeding dystonia. Most importantly, testing for elevated serum CK seems to be a good and cheap screening test in suspected MLS with a sensitivity of 97%.

Since human X-chromosomes are inactivated randomly in the blastocyst stage with approximately 58–84 cells [41], milder phenotypes of MLS in women are plausible [42]. Six women with chorea have been briefly described in the literature, yielding a ratio of 1:10 versus men.

This systematic review reinforced our view that it is unlikely that a woman should develop a new phenotype with not yet described vertical gaze palsy in the context of MLS when clinical and MRI findings for MLS are absent but indicate PSP. Vertical gaze impairment has only been described in chorea-neuroacanthocytosis to date. The patient was 73 years old at the age of diagnosis of PSP which is the common age of diagnosis of PSP which makes a sporadic case of PSP more likely in the presence of this main risk factor [43].

Limitations are the publication bias of more interesting cases, the point of view of the cited authors, who were sometimes focusing on hematological or genetic aspects, and missing laboratory results in the reports. Confounders such as side effects of medication may have been present but not reported. This retrospective approach cannot establish the life expectancy after symptom onset.

Compared to the literature, we refined the prevalence of signs and symptoms by describing the clinical characteristics of 65 male and 11 female patients. The findings are similar to known publications such as Danek et al. 2001[3]. Our relatively low findings of hepato- and splenomegaly are probably due to a lack of investigations of hepatosplenomegaly and different handling of missing values.

To conclude, diagnosis of MLS is suspected in cases with the typical clinical phenotype, absent tendon reflexes and elevated serum levels of creatine kinase. Immunohematological examination demonstrates the absence of Kx- and weakened Kell red blood surface antigens, the so-called McLeod blood group phenotype, and proves the diagnosis of MLS. Diagnosis can be secured by molecular genetic testing of the XK gene, where variations (mainly deletions or nonsense mutations) lead to a truncated XK protein, which is a transmembrane protein of red blood cells and other tissues.[4].

Any genetic carrier status can be an anchor that influences our clinical evaluation. The disease is always expected to appear one day. The conclusion that new symptoms are due to the known alteration is readily made due to anchoring bias, which is a cognitive bias influencing our decision by a reference point such as a genetic carrier status. [44].

Unusual in this case were the occurrence of a rare disease in its typical form and its delayed diagnosis due to the stigma of a rare genetic carrier status. If a PSP phenotype is more prevalent in MLS carriers, the carrier status may constitute a risk factor. We hereby reported the first PSP phenotype in an MLS carrier.

## Data Availability

The Microsoft Excel table that was created summarizing all cited papers is available on https://github.com/Andi-Braun/ReviewMcLeodExcel.git.

## Abbreviations

ALT: Alanine transaminase
AST: Aspartate transaminase
ChAc: Chorea-neuroacanthocytosis
ENMG: Electroneuromyography
FDG-PET: Fluorodeoxyglucose positron emission tomography
FLAIR: Fluid attenuated inversion recovery
Kx: Kell antigen associated with MLS
LDH: Lactate dehydrogenase
MLS: McLeod Syndrome
MRI: Magnetic resonance imaging
PSP: Progressive supranuclear palsy
VPS13A: Vacuolar protein sorting 13 homolog A
